# Inhibitory Effect of Propolis on Platelet Aggregation In Vitro

**DOI:** 10.1155/2017/3050895

**Published:** 2017-10-10

**Authors:** Yun-Xiang Zhang, Ting-Ting Yang, Liu Xia, Wei-Fen Zhang, Jia-Fu Wang, Ya-Ping Wu

**Affiliations:** ^1^Department of Pathology, Weifang People's Hospital, Weifang, Shandong Province 261041, China; ^2^School of Pharmacy, Weifang Medical University, Weifang, Shandong Province 261053, China; ^3^Department of Ophthalmology, Weifang Medical University, Weifang, Shandong Province 261053, China; ^4^Department of Pathology and Pathophysiology, Taishan Medical University, Taian, Shandong Province 271016, China; ^5^Departments of Haematology of University Medical Center, Utrecht University, 3508GA Utrecht, Netherlands

## Abstract

Platelet hyperactivity plays an important role in arterial thrombosis and atherosclerosis. The present study was aimed to investigate the effects of different extracts of propolis and components of flavonoids on platelet aggregation. Platelet-rich plasma was prepared and incubated in vitro with different concentrations of the tested extracts and components of flavonoids. Platelets aggregation was induced by different agonists including adenosine diphosphate (ADP, 10 *μ*M), thrombin receptor activator peptide (TRAP, 50 *μ*M), and collagen (5 *μ*g/mL). At 25 mg/L to 300 mg/mL, the water extract propolis (WEP) inhibited three agonists-induced platelet aggregations in a dose-dependent manner. The flavonoids isolated from the propolis also showed markedly inhibited platelet aggregation induced by collagen, ADP, and TRAP, respectively. The components including caffeic acid phenethyl ester (CAPE), galangin, apigenin, quercetin, kaempferol, ferulic acid, rutin, chrysin, pinostrobin, and pinocembrin and their abilities of inhibiting platelet aggregation were studied. It was concluded that propolis had an antiplatelet action in which flavonoids were mainly implicated.

## 1. Introduction

Nowadays, cardiovascular diseases (CVD) are the leading cause of morbidity and mortality worldwide [1–3] and bring a huge burden to the world economic development and people's living standard. It has been widely known that platelets play important roles in both hemostasis and pathogenesis of CVD such as acute coronary syndrome [4]. Platelet inhibition has shown improved short- and long-term clinical outcomes for CVD patients. However, increased bleeding risk and the high rates of recurrent ischemic events could not be ignored [5]. Furthermore, the activation of platelets was also related to circulation and vascular damage in patients with hypertension and diabetes [6]. Antiplatelet drugs used clinically to treat and prevent coronary syndromes and stroke are accompanied by a variety of side effects such as thrombocytopenia, hemorrhage, gastric ulcers, and therapeutic resistance [7, 8]. More safe and effective antiplatelet drugs would be urgently needed based on the current situation.

In recent, natural products and alternative medicine are now getting significant attention. Propolis, a complex mixture containing various compounds, such as flavonoids, terpenes, β-steroids, aromatic aldehydes, and alcohols, is a plant-derived substance collected from plant materials by honeybees [9, 10]. Propolis is extensively used in food and beverages to improve health because of its unique pharmacological activities including antimicrobial, antioxidant, immunomodulatory, hepatoprotective, antitumor, and cardioprotective effects [11–21]. Recently, Liu and his colleagues put forward that chrysin in propolis performs antiplatelet activity via inhibiting platelet alphaIIbbeta3-mediated signaling pathway [22]. In addition, evidence data showed that propolis coated Co-Cr could significantly reduce adhesion of platelets [23]. Many studies demonstrated that the propolis components were varied in different geographic and climatic zones [24]. Thus, the aim of the present study was to examine the effects of propolis extracts on human platelet aggregation in vitro and to identify the nature of the compounds responsible for the antiplatelet activity.

## 2. Materials and Methods

### 2.1. Materials

Propolis sample was obtained from Taishan fir in the autumn. Adenosine diphosphate (ADP) was purchased from Arkray (Aggrepack, Japan). Collagen was purchased from NYCOMED (Kollagenreagens Horm, Austria). Thrombin receptor activator peptide (TRAP) was purchased from Bachem (Germany). Caffeic acid phenethyl ester (CAPE), galangin, acacetin, and pinostrobin were purchased from Sigma Chemical Co. (USA).

### 2.2. Preparation of Water Extract of Propolis (WEP) and Flavonoid

According to previous research [25], a certain amount of dried propolis sample frozen at −20°C was dissolved in 30 mL of distilled water at 60°C for 7 h. The crude extract was filtered, then centrifuged at 28000 rpm for 30 min, and the supernatants were concentrated under reduced pressure to produce the WEP. Flavonoid was obtained from another 10.0 g dried propolis dissolved in 300 mL 70% ethanol 7 h at room temperature using the same method [26].

### 2.3. Component Analysis of WEP and Flavonoid

For the components of WEP and flavonoid, a ZQ-4000 HPLC apparatus (Waters, USA) was used. The chromatographic separation was achieved using a Waters Symmetry C18 column (4.6 mm I.D. × 150 mm, 3.5 *μ*m), with the column oven temperature maintained at 25°C. The mobile phase consisted of acetonitrile and 2% acetic acid. The mobile phase flow rate was 0.2 mL/min, and UV absorbance was monitored at 280 nm. Diluted standard solutions of galangin, CAPE, apigenin, quercetin, kaempferol, ferulic acid, rutin, chrysin, pinostrobin, and pinocembrin were analyzed in the same HPLC conditions, and furthermore, the calibration of the detector response was done. Quantification of the main bioactive compounds from WEP and flavonoid was based on the calibration curves. The results were confirmed by the Department of Agriculture Bee Products Quality Supervision and Inspection Center (Beijing).

### 2.4. The Platelet Aggregation Tests

Human platelet suspensions were prepared as previously described [27]. In this study, human volunteers had been given informed consent. In brief, blood was collected from healthy human volunteers who had taken no medicine during the preceding 2 weeks and was mixed with 3.4% Na-citrate (9 : 1, vol/vol). The blood was centrifugated at 1100 rpm for 15 min at 20°C. Then, platelet-rich plasma (PRP) was collected with pipette and stored at room temperature. The tubes containing PRP were centrifuged at 3000 rpm for 10 min at 20°C to obtain the platelet-pool plasma (PPP). After a platelet count (Sysmex SE-9000, Kobe, Japan), a standard concentration of 200 × 109/L was obtained by diluting PRP with PPP. The turbidimetric method was applied to measure platelet aggregation stimulated by the various kinds of agonists using an aggregometer [28]. Previous study mentioned different flavonoid components present in propolis (CAPE, galangin, acacetin, and pinostrobin) were able to inhibit platelet aggregation, so CAPE, galangin, acacetin, and pinostrobin were tested in our study [29]. All aggregation tests were performed within 2 h after isolation and carried out in triplicate. Each person had his/her own 0% PRP and 100% PPP aggregation calibration. Before aggregation was started, standard platelet concentration was incubated in solutions of different concentrations, including WEP (300 mg/L, 100 mg/L, 25 mg/L), flavonoids (400 mg/L, 100 mg/L, 25 mg/L), CAPE (352 *μ*M, 88 *μ*M, 6 *μ*M), galangin (370 *μ*M, 185 *μ*M, 46 *μ*M), acacetin (352 *μ*M, 176 *μ*M, 44 *μ*M), and pinostrobin (370 *μ*M, 92 *μ*M, 23 *μ*M) for 30 min at the room temperature, then prewarmed at 37°C for two minutes. The aggregation was induced by ADP, collagen, or TRAP, respectively. The extent of aggregation was expressed in light transmission units.

### 2.5. Statistical Analysis

The results were shown as mean ± standard deviations, and each experiment was performed in triplicate. Statistical analysis was carried out using SPSS19.0, and differences were considered to be significant at a level of *P* < 0.05.

## 3. Results and Discussion

### 3.1. Chemical Components of Propolis

Flavonoids are the major constituents of propolis and contribute greatly to the pharmacological activities of propolis. Usually, the quality of propolis was based on flavonoids quality [30]. The contents of flavonoids depend on the harvest region because the characteristics of propolis are influenced by the local plant varieties and weather [31]. Report of component analysis of WEP and flavonoids was shown in Table 1. The contents of CAPE were the highest in the WEP, followed by galangin, ferulic acid, quercetin, kaempferol, and apigenin. Pinostrobin was the most abundant chemical compound in flavonoids, followed by galangin, chrysin, CAPE, rutin, kaempferol, pinocembrin, apigenin, quercetin, and ferulic acid.

### 3.2. The Inhibition Effect of Different Components on Platelet Aggregation Induced by Different Agonists

**Figure 1:**
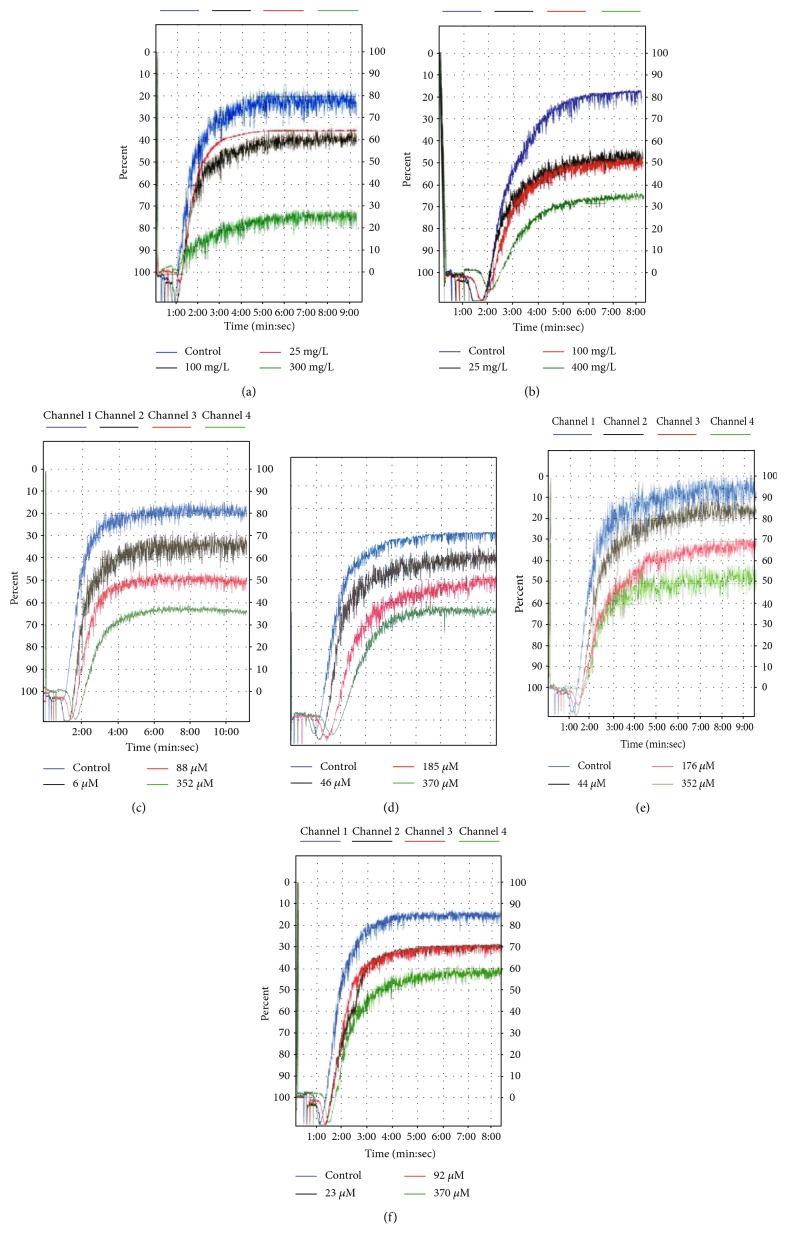
Original tracings showing the dose-dependent inhibitory effect of different extracts including WEP (a) and flavonoids (b) and pure components including CAPE (c), galangin (d), acacetin (e), and pinostrobin (f) on collagen-induced platelet aggregations in vitro.

**Figure 2:**
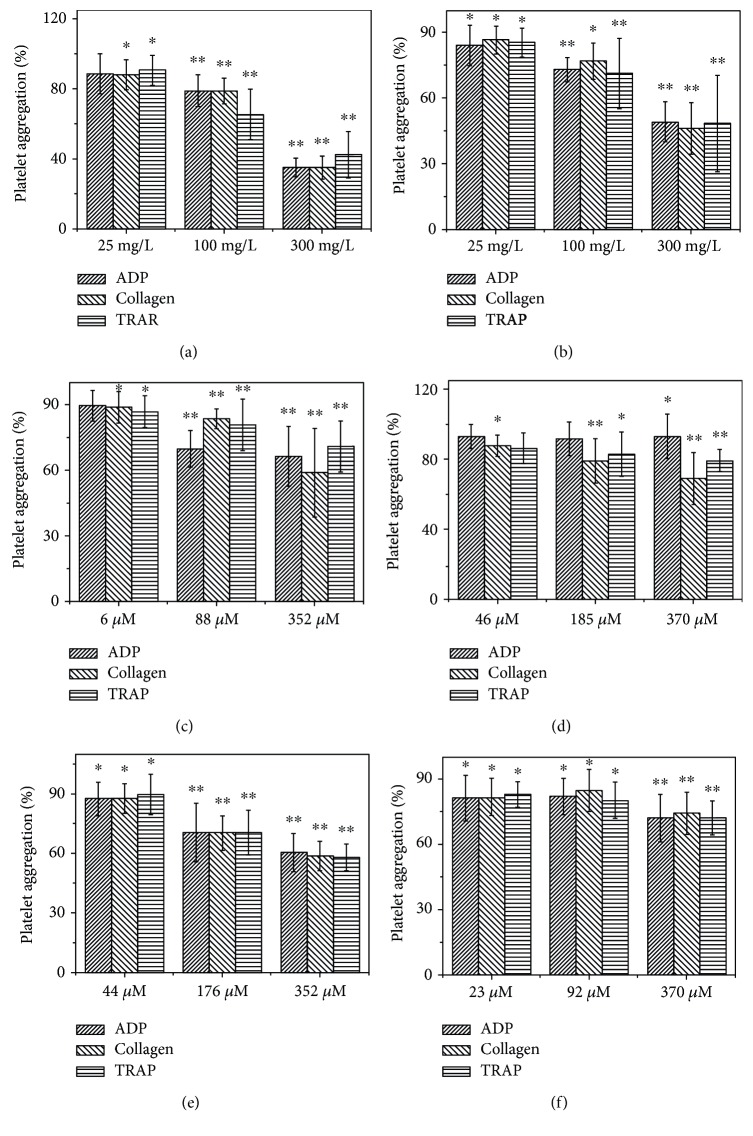
Inhibitory effect of different extracts including WEP (a) and flavonoids (b) and pure components including CAPE (c), galangin (d), acacetin (e), and pinostrobin (f) on platelet aggregation induced by different agonists including ADP, collagen, and TRAP, respectively. ^∗^Compared with the control (*P* < 0.05) was observed. ^∗∗^Compared with the control (*P* < 0.01) was observed. Data are represented as the mean ± SD (*n* = 3).

Results of the platelet aggregation tests were shown in Figures 1 and 2. The results showed that the crude WEP inhibited platelet aggregation in a dose-dependent manner, which might be stimulated with different agonists including ADP, collagen, and TRAP. For instance, 300 mg/L WEP significantly decreased platelet aggregation induced by ADP, collagen, and TRAP to 35.00 ± 5.34%, 35.00 ± 6.61%, and 42.33 ± 13.24% (*n* = 3; *P* < 0.01), respectively, indicating that propolis contains compounds having antiplatelet aggregation activity. This effect might be attributed to polar compounds such as flavonoids and polyphenols.

Flavonoids inhibited the three agonist-induced platelet aggregations in a dose-dependent manner, and the percent of inhibition were 53.11 ± 1.90%, 51.12 ± 9.30%, and 51.98 ± 22.20% at concentrations of 400 mg/L, respectively. This finding was consistent with previous studies showing the antiplatelet aggregation activity of flavonoids in vitro [32] and in vivo [33]. We also test four purified components, including CAPE, galangin, acacetin, and pinocembrin. All test dose CAPE added to the PRP platelet aggregation induced by collagen and TRAP significantly decreased (*P* < 0.05), and the most antiplatelet aggregation effect occurred in the collagen-included platelet aggregation test group with an inhibition of 58.82 ± 4.51% at 176 *μ*M. Significant inhibition was not detected in the ADP-included platelet aggregation test group when adding 6 *μ*M CAPE. When 46 *μ*M or more galangin was added to PRP, significant inhibition was detected in the collagen-included platelet aggregation test group in a dose-dependent manner. Galangin was not able to inhibit ADP-induced platelet aggregation until 370 *μ*M galangin had been added. Acacetin could significantly inhibit platelet aggregation induced by ADP, collagen, and TRAP at concentrations of 44, 176, and 352 *μ*M in the concentration-dependent manner (*P* < 0.05). The antiplatelet aggregation activity of pinostrobin isolated from propolis (23 *μ*M, 92 *μ*M, and 370 *μ*M) was evaluated upon ADP-, collagen-, and TRAP-induced aggregation. As shown in Figures 1(f) and 2(f), the platelet aggregation could be suppressed by pinostrobin at concentrations of 23–370 *μ*M (*P* < 0.005). CAPE appeared to be more effective inhibitors than galangin and acacetin. This difference in potency could be explained by their chemical structures [34].

The isolated flavonoids markedly inhibited platelet aggregation induced by ADP, TRAP, and collagen. The inhibition might be introduced by holding back fibrinogen binding to its platelet membrane receptor (glycoprotein (GP) IIb-IIIa), which is a final and common pathway of platelet aggregation. It was reported that the flavonoid phloretin diminished ADP and TRAP-stimulated expression of the activated form of the GPIIb/IIIa complex and reduced platelet aggregation stimulated by ADP [30]. Several other studies had shown that flavonoids inhibited platelet function through a multitude of mechanisms including decreasing phospholipase C activity [31], scavenging of reactive oxygen species such as superoxide anion, and inhibiting cyclic nucleotide phosphodiesterase and thromboxane A2 synthesis [32].

## 4. Conclusion

The main components of propolis were galangin, CAPE, apigenin, quercetin, kaempferol, ferulic acid, rutin, chrysin, pinostrobin, and pinocembrin. The WEP and flavonoids extracted from propolis exhibited dose-dependent inhibitory effects on platelet aggregation induced by different agonists including ADP, collagen, and TRAP, respectively. CAPE, galangin, and pinostrobin might be mainly implicated. Further studies are necessary to clarify the mechanism of platelet inhibition.

## Figures and Tables

**Table 1:** The components of WEP and flavonoids.

Compounds of propolis	Concentration in WEP (mg/L)	Concentration in flavonoids (mg/g)
Galangin	4477.09	37.24
CAPE	6329.74	5.55
Apigenin	305.76	0.34
Quercetin	2681.01	0.17
Kaempferol	897.80	1.79
Ferulic acid	3593.05	0.06
Rutin	—	3.56
Chrysin	—	13.94
Pinostrobin	—	95.52
Pinocembrin	—	0.84
